# HIV clinical stages and lower extremity arterial disease among HIV infected outpatients in Burundi

**DOI:** 10.1038/s41598-021-87862-z

**Published:** 2021-04-15

**Authors:** Ileana Desormais, Deo Harimenshi, Théodore Niyongabo, Philippe Lacroix, Victor Aboyans, Pierre Marie Preux

**Affiliations:** 1 Department of Vascular Surgery and Vascular Medicine, Dupuytren University Hospital, Limoges, France; 2 CHU Limoges, IRD, U1094 Tropical Neuroepidemiology, Institute of Epidemiology and Tropical Neurology, GEIST, INSERM, Univ. Limoges, Limoges, France; 3 Department of Referral Centre of HIV, Bujumbura, Burundi; 4 Department of Cardiology, Dupuytren University Hospital, Limoges, France

**Keywords:** Cardiovascular diseases, Infectious diseases

## Abstract

Chronic disease of people living with human immunodeficiency virus (HIV) infection are now approaching those of the general population. Previous, in vitro studies shown that HIV causes arterial injuries resulting in inflammation and atherosclerosis but direct relationship between HIV infection clinical stages and lower extremity arterial disease (LEAD) remain controversial. No study assessed, with an accurate method, both the prevalence of LEAD and the influence of HIV severity on LEAD in HIV outpatients in Central Africa. A cross-sectional study was conducted among 300 HIV-infected outpatients, aged ≥ 40 years in Bujumbura, Burundi. All patients underwent ankle-brachial index (ABI) measurement and LEAD was diagnosed by ABI ≤ 0.9. The prevalence of LEAD was 17.3% (CI 95% 13.2–22.1). The mean age was 49.6 ± 7.1 years. On multivariable analysis, factors associated with LEAD were hypertension (OR = 2.42; 95% CI 1.10–5.80), and stage IV HIV clinical infection (OR = 4.92, 95% CI 1.19–20.36). This is the first study performed on a large HIV population in Central Africa, reporting high LEAD prevalence. It underlines the influence of HIV infection on peripheral atherosclerosis at latest clinical stages and the need for LEAD screening in HIV-infected patients.

## Introduction

During the last decades, the introduction of combined antiretroviral therapy (ART) has led to remarkable reduction in HIV/AIDS-related mortality. Nowadays, the life expectancy and chronic disease of people living with HIV infection (PLWH) are approaching those of the general population^1^. Among HIV-infected patients, particularly those who receive ART, several atherogenic metabolic disorders including dyslipidaemia, lipodystrophy and insulin resistance are reported^2–4^. Previous studies have also shown that HIV causes direct injury to the arterial wall resulting in inflammation and atherosclerosis^5^ and that cardiovascular disease (CVD) became one of the main causes of morbidity and mortality among PLWH^6,7^. Multiple observational cohort studies^6,8–11^ have demonstrated elevated rates of coronary artery disease in HIV-infected patients, with an approximate 1.5 to twofold increased relative risk as compared to controls.

A recent meta-analysis estimated that HIV infection confers a 61% increased risk of cardiovascular mortality. Moreover, when limited to HIV-infected patients on ART, the relative-risk of CVD increases twofold as compared to no-HIV and 1.5-fold compared to treatment-naive HIV-infected patients^12^.

Lower extremity arterial disease (LEAD) is the second most common form of cardiovascular disease after coronary artery disease. Nevertheless, few specific studies assessed the prevalence of LEAD among PLWH. Available data are issued from studies in hospitalized patients^13,14^, urban population^15^ or age limited populations, using heterogenous definition of LEAD^14,16^ or focusing on specific clinical LEAD stages^17^. Results are controversial^18,19^ as study populations are not comparable. None of the studies reported any precise, accurate correlation between clinical stages of HIV disease and the prevalence of LEAD in African HIV population.

Within its 11 million inhabitants, Burundi has one of the highest prevalence of PLWH worldwide. In 2016, this country had 2200 new HIV infections and 2900 Acquired Immuno Deficiency Syndrome—related deaths. There were 84,000 people living with HIV, among whom 61% were accessing ART. In Burundi, survey and treatment of HIV infected patients are mainly dispensed in HIV centres in Bujumbura capital.

Due to the paucity of data and controversial results on the prevalence of LEAD in PLWH, our main objective was to determine, using accurate methods in line with the current guidelines, the prevalence of LEAD in HIV outpatients in Burundi and particularly determine the influence of HIV disease clinical stages.

## Methods

During 3 months a cross-sectional study, dealing with rural and urban patients, was conducted in four HIV centres in Bujumbura capital (BURUNDI). The aim of our study was to determine the prevalence of LEAD among HIV infected subjects, describe the associated cardiovascular risk factors and analyse the association between HIV clinical stages and LEAD.

### Study design

The study population included, successively, all HIV infected outpatients (urban and rural population), aged ≥ 40 years. Refusal to participate was the exclusion criterion. Informed written consent was obtained before inclusion. Data were collected through a face-to-face interview, a clinical examination and laboratory tests.

### Ethics

The study obtained the approval of the National Ethics review Committee (NEC) from Burundi (Comité National d’Ethique pour la protection des êtres humains sujets de la recherche biomedicale et comportementale, Burundi).

The research reported in the paper was undertaken in compliance with Helsinki Declaration.

### Data collection and definitions

The detailed administered questionnaire, adapted from World Health Organization (WHO) STEPS tools^20^ included demographic and clinical characteristics: age, gender, lifestyle factors (smoking, alcohol intake), medical history (hypertension, diabetes, cardiovascular disease), current medication (antihypertensive agents, anti-diabetic drugs), past- and current occupation, education (school cycles completion), marital status. Smoking status was classified as never, past (smoking cessation since more than 1 year prior to the survey) or current smoking. Alcohol drinking was assessed based on the frequency and amount of alcohol intake and was categorized as never, occasional (less than 5 days/week) and regular (more than 5 days/week). Body-mass index (BMI) was calculated as weight/height^2^ and categorized according to the WHO as: underweight (< 18.5 kg/m^2^), normal weight (18.5–24.9 kg/m^2^), overweight (25–29.9 kg/m^2^), and obese (≥ 30 kg/m^2^). Pulse rate, systolic and diastolic blood pressure (average of two measures for each arm at 1-min interval) were recorded. Hypertension was defined in case of self-reported ongoing antihypertensive treatment and/or systolic blood pressure ≥ 140 mm Hg and/or diastolic blood pressure ≥ 90 mm Hg^21^. Diabetes was defined according to self-reported diabetes medication or in case of elevated capillary blood glucose level (Accu-Chek Performa, Roche) above 126 mg/dL if the fasting period > 2 h or above 200 mg/dL in non-fasting participants. Furthermore, blood samples were used to determine high-sensitivity C-reactive protein (hs-CRP), cholesterol levels, triglyceride levels, high-density lipoprotein (HDL) and low-density lipoprotein (LDL). Renal dysfunction was defined as end stage renal disease with dialysis, or a glomerular filtration rate (GFR) < 60 mL/minute/1.73 m^2^ calculated according the Cockcroft**-**Gault formula.

The ABI was measured using a manual cuff and a hand-held Doppler device (Super Dopplex II, Huntleigh Technology PLC, Luton, UK) to determine, on both arms and legs, the systolic blood pressure in the posterior tibial and dorsalis pedis and brachial arteries. Measurements were done in accordance to the American Heart Association guidelines. The ABI for each leg was calculated as the ratio between the higher of the two systolic blood pressure measurements in each leg (posterior tibial or dorsalis pedis) and the higher of the two systolic blood pressure measurement in the arms. The value of the limb with the lowest ABI was used for each patient. An ABI ≤ 0.90 defined LEAD, and an ABI ≥ 1.40 defined high ABI^22^.

Presumed duration of HIV infection, duration of antiretroviral therapy (years), HIV detectable load (≥ 200 copies/ml), CD4 + T cell count (cells/ml) were revealed. HIV clinical stages (stage I to IV) were defined following the WHO consolidated guidelines on clinical staging of HIV disease in adults, adolescents and children 2016^23^.

### Statistical analysis

The data were analysed with the statistical program Stata 12 (StataCorp, College Station, TX). The Student t-test or the Mann–Whitney test were used for the comparison of the continuous variables and the Chi-square test or Fisher exact test for the comparison of the categorical variables. In order to determine independent risk factors associated with LEAD, a multivariable logistic regression model including backward stepwise procedure was performed. The level of significance for all the statistical analyses was set at *P* < 0.05 and interactions were checked. Some variables as age and sex or “traditional CV risk factors” (current smoking, diabetes, elevated plasma cholesterol level) were forced in the multivariable model estimating the association between LEAD and clinical HIV stages.

## Results

**Figure 1:**
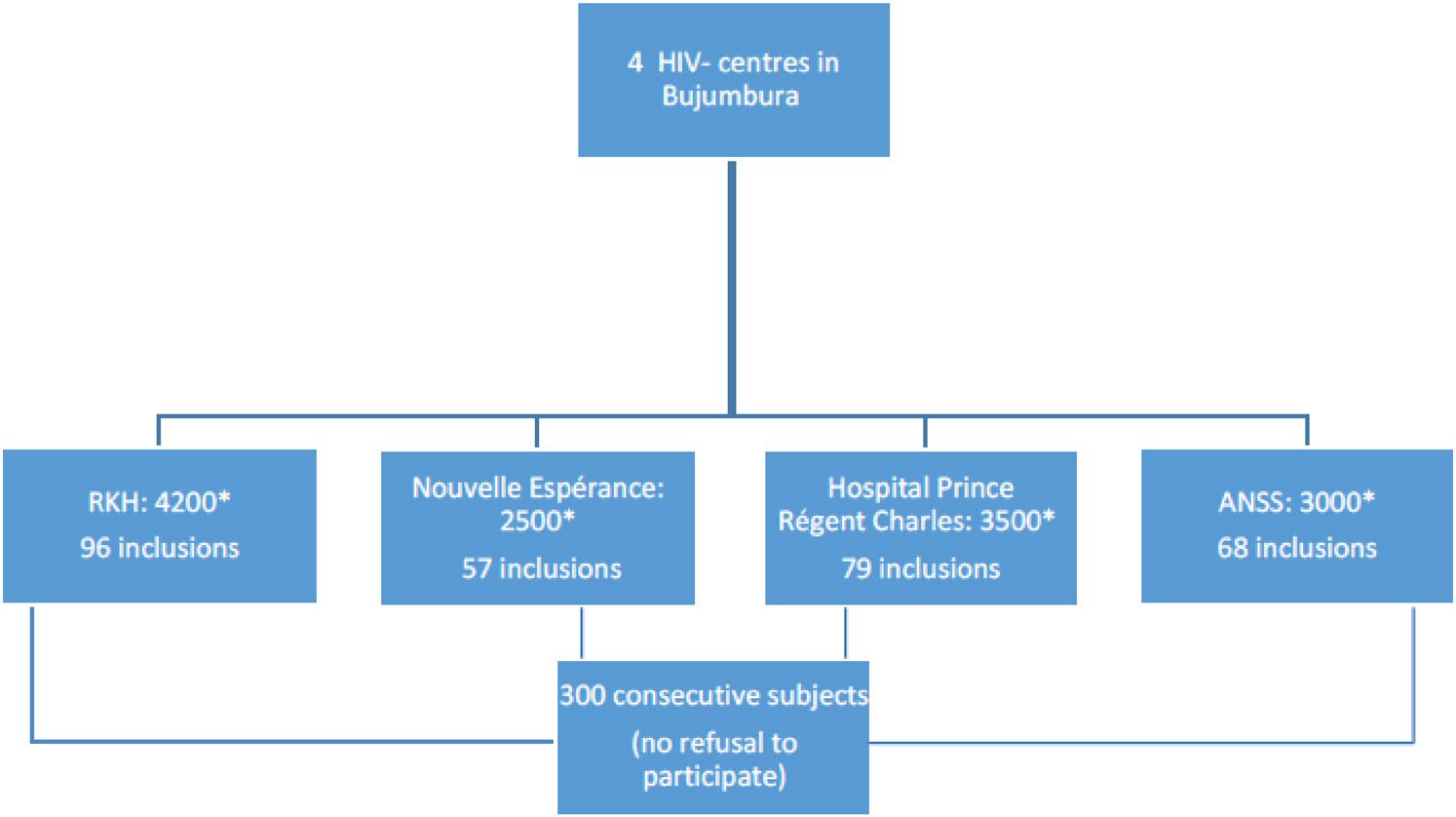
Flow chart. *ANSS* Burundi National Association for people living with HIV, *RHK* Roy Khaled Hospital, *followed HIV infected subjects/year.

Three hundred consecutive HIV-infected outpatients (81.2% women), in four centres in Bujumbura (43% rural subjects) were included in the study. No subject declined participation. The flow chart is presented in Fig. 1.

The prevalence of low ABI (≤ 0.9) was 17.33% (n = 52) while 6.9% (n = 13) had incompressible arteries. The mean age of LEAD subjects was 49.5 ± 7.1 years. There was no significant age difference between patients with- or without LEAD (*P* = 0.14). General characteristics of the population are presented in Table 1. Only 6 subjects were active smokers (2.7%), no past smokers were registered. Possibly due to the small size of the sample, no significant difference between LEAD and no LEAD subjects was identified.

**Table 1:** Comparison of cardiovascular risk factors.

	n (%) 287 (100)	LEAD		*P* value
		n (%) 52 (18.1)	n (%) 235 (81.9)	
Age (years)	50.9 ± 7.3^a^	49.5 ± 7.1^a^	51.2 ± 7.4^a^	0.14
Sex (female)	233 (81.2)	45 (85.5)	118 (80)	0.27
Hypertension	43 (14.9)	10 (19.2)	33 (14)	0.89
Diabetes mellitus	17 (5.9)	4 (7.7)	13 (5.5)	0.52
Current smoking	6 (2.1)	2 (3.4)	4 (1.7)	0.29
Chronic kidney disease	61 (21.2)	13 (25)	48 (20.4)	0.53
Elevated plasma triglyceride level	83 (28.9)	18 (38.6)	65 (27.6)	0.31
Elevated plasma cholesterol level	14 (4.9)	1 (1.9)	13 (5.5)	0.47
**Body mass index (Kg/m**^2^**)**				0.33
< 18	36 (12.5)	8 (15.38)	28 (11.91)	
18–24.9	143 (49.8)	27 (51.38)	116 (49.3)	
25–29.9	70 (24.3)	8 (15.38)	62 (26.38)	
≥ 30	38 (13.24)	9 (17.31)	29(12.34)	

^a^Mean ± SD; LEAD: Lower extremity arterial disease, Elevated cholesterol level > 5.7 mmol/l, Elevated plasma triglyceride > 1.72 mmol/l.

Patients with- and without LEAD had similar other CV associated risk factors and comorbidities. In order to identify factors associated with LEAD, 13 patients with ABI ≥ 1.40 were excluded from our analysis. Characteristics of excluded subjects were similar to those of the study population.

The mean CD4 count was 430.7 cells/mL and 8.4% (n = 24) of the subjects had detectable HIV RNA viral loads. The presumed duration of HIV infection was 9.91 years and the duration of ART 7.53 years. HIV clinical stages weren’t similar in both population (*P* = 0.04) (Table 2).

**Table 2:** Comparison of clinical characteristics related to HIV.

Characteristics	Overall	Lower extremity arterial disease		
		Yes	No	*P*
	n (%) 287 (100)	n (%) 52 (18.1)	n (%) 235 (81.9)	
HIV load (≥ 200 copies/ml)	24 (8.4)	7 (29.2)	17 (70.8)	0.16
Estimated duration of HIV (years)	9.91 ± 4.60^a^	9.36 ± 4.56^a^	10.03 ± 4.64^a^	0.34
**HIV clinical stage**				
Stage 1	49 (17.1)	8 (16.3)	41 (83.7)	0.66
Stage 2	93 (32.4)	11 (11.8)	82 (88.2)	0.53
Stage 3	130 (45.3)	27 (20.8)	103 (79.2)	0.45
Stage 4	15 (5.2)	6 (40)	9 (60)	**0.04**
Duration on ART (years)	7.53 ± 3.35^a^	7.53 ± 3.44^a^	7.53 ± 3.33^a^	0.66
**CD4 + T cell count, cells/mL**				
< 200	15 (5.2)	4 (26.6)	11 (73.4)	0.07
200–500	121 (42.2)	21 (17.6)	100 (82.4)	0.06
> 500	151 (52.6)	27 (17.9)	124 (82.1)	0.12

Bold value indicates level of significance for statistical analyses set at *P* < 0.05.

*ART* antiretroviral therapy.

^a^Mean ± SD.

**Figure 2:**
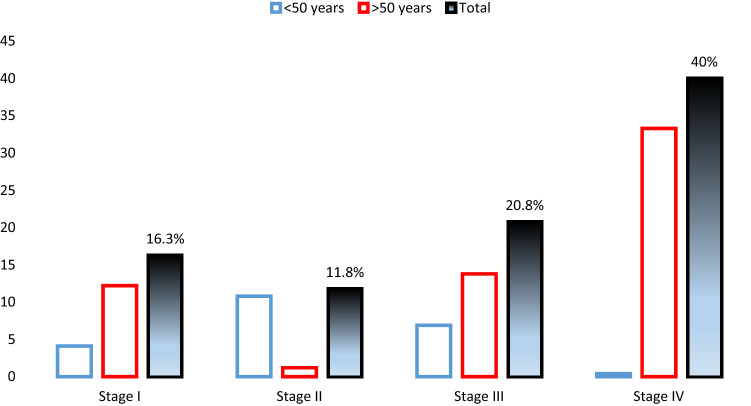
Lower extremity arterial disease prevalence according to the HIV clinical stages (I, II, III, IV).

The prevalence of LEAD was increasing with the latest clinical HIV-stages and was higher in patients aged 50 or more (Fig. 2).

The HIV clinical stage IV (OR 3.41, 95% CI 1.04–13.46, *P* = 0.04) was associated with LEAD even after adjustment to all CV risk factors, CV morbidities and HIV infection characteristic. Results are summarised in Table 3. After multivariable analysis, factors associated with LEAD were hypertension (OR = 2.42; 95% CI 1.10–5.80; *P* = 0.04), and stage IV of HIV infection (OR = 4.92, 95% CI 1.19–20.36; *P* = 0.03). The association between LEAD and current smoking or HIV load did not reach statistical significance (*P* = 0.09).

**Table 3:** Associated Lower extremity arterial disease (LEAD) factors.

Characteristics	Univariate logistic regression			Multivariable logistic regression		
	Odds ratio	CI 95%	*P*	Odds ratio	CI 95%	*P*
Age (per year)	0.96	[0.92–1.01]	0.14	0.90	[0.89–0.99]	**0.03**
Sex (female)^a^	1.60	[0.68–3.79]	0.27	1.60	[0.61–4.15]	0.33
Hypertension^a^	1.34	[0.63–2.85]	0.43	2.42	[1.10–5.80]	**0.04**
Current smoking^a^	2.31	[0.41–12.9]	0.34	5.31	[0.76–36.90]	0.09
Elevated plasma cholesterol level^a^	0.33	[0.04–2.61]	0.27	0.21	[0.23–1.77]	0.15
HIV load (≥ 200 copies/ml)	5	[0.78–5.09]	0.14	2.49	[0.86–7.13]	0.09
**HIV clinical stage**						
2	0.68	[0.27–1.94]	0.53	0.71	[0.24–2.04]	0.52
3	1.39	[0.59–3.30]	0.45	1.76	[0.68–4.53]	0.23
4	3.41	[1.04–13.46]	**0.04**	4.92	[1.19–20.36]	**0.03**

Bold value indicates level of significance for statistical analyses set at *P* < 0.05.

^a^Forced variables.

## Discussion

The major finding of our study is the high prevalence of LEAD in mid-aged HIV-infected outpatients, especially at the latest HIV clinical stages. To our knowledge, this is the first study on a large sample of HIV infected outpatients in Central Africa that accurately establish a clear relationship between clinical HIV stages and LEAD prevalence. Also, our study is the first one dealing with urban and rural HIV outpatients on the African continent.

Previous studies of LEAD among HIV-infected patients have reported prevalence ranging from 1 to more than 10% (Table 4). The prevalence of LEAD in the general population has been estimated between 2 and 4% in two large studies in young adults in USA^24,25^. In a cross-sectional hospital-based study in black Africans, the LEAD prevalence was estimated at 6.9%^14^. A recent Nigerian study reported no difference of LEAD prevalence between virologically-suppressed individuals and controls^19^. Both studies defined LEAD as an ABI < 0.9 and had methodologic bias. In our study, the prevalence of LEAD defined by an ABI ≤ 0.9 was 17.3% for a mean age of 49 ± 7.1 years. This prevalence is higher than expected in HIV mid aged patients and is comparable to the prevalence of LEAD in African elderly (> 70 years) general population^26^. These very different results reported in the literature, are due to the limited samples, the selection bias (hospital recruitment, exclusively asymptomatic subjects, young age, occidental population) and heterogenous LEAD diagnosis methods. Also, beyond HIV infection, Caucasian and African populations are difficult to compare. Prior studies have consistently demonstrated higher prevalence of LEAD among African Americans or African native subjects as compared to Caucasians^26–28^. This racial difference in LEAD prevalence remains independent and is only modestly attenuated by adjustment for traditional CVD risk markers as age, diabetes, smoking, plasma lipids and hypertension^29,30^. As many of these factors are very common in HIV patients receiving antiretroviral therapy a high prevalence of LEAD might be expected in PLWH^31,32^. Nevertheless, correlation between CV risk factors and LEAD in PLWH is inconsequent and varies from no association at all to different correlations, between hypertension, age, diabetes and/or smoking and LEAD, from one country to another. This might be due not only to the selection bias but also to different lifestyle factors. As tobacco is less common in African women, the lack of association between LEAD and smoking in our study is surprising but not unique in the literature. Four other studies, specifically undertaken in the elderly general population in Central Africa, also failed to find this association^26,27,33, 34^.

**Table 4:** Last decade cross-sectional studies on LEAD prevalence in HIV infected subjects.

Author study	Country	Study population	Recruitment	LEAD definition	LEAD prevalence (%)
Salas D 2010	Spain	231	Urban outpatients	ABI ≤ 0.90	1.3
Qaqa AI 2012	USA	173	Urban outpatients	ABI < 0.90	13.9
Knudsen AD 2015	Denmark	102	Urban clinic	ABI ≤ 0.90	1
Knudsen AD 2018	Denmark	908	CGPD urban	ABI ≤ 0.90	12
Kamden F 2018	Cameroun	144	Hospital	ABI < 0.90	6.9
Agu C 2019	Nigeria	200	Hospital	ABI < 0.90	14.6
Our Study	Burundi	300	Urban and rural outpatients	ABI ≤ 0.90	17.3

*ABI* ankle brachial index, *CGPD* Copenhagen general population study, *LEAD* lower extremity arterial disease.

Among the HIV related factors, only HIV clinical stage IV was associated with LEAD (OR = 4.92, 95% CI 1.19–20.36; *P* = 0.03). This association has also been described, in hospital Spanish and Cameroon populations. These studies had either important LEAD diagnosis bias^14^ or exclusively focused on asymptomatic LEAD subjects^17^. Our results accurately analysed the association between HIV clinical stage and LEAD in urban and rural HIV outpatients and indicate that the severity of HIV infection could influence the arteries injury.

The role of the HIV infection as a LEAD risk factor was also prospectively established on over 90,000 participants (29,291 HIV infected subjects and 62,996 sex- and age-matched controls) in the Veterans Aging Cohort Study^35^. The study had nevertheless been designed for the analyse of the role of alcohol use and abuse in determining clinical outcomes in Americans. Also, no LEAD prevalence in PLWH was reported.

After adjusting for age, sex, race and cardiovascular risk factors including LDL cholesterol, triglycerides, diabetes, smoking, hypertension, obesity, impaired kidney function, hepatitis C infection, and alcohol or cocaine abuse, the investigators found an increased risk of LEAD in subjects with CD4 cell counts below 500 cells/mm^3^. This increased risk was also reported with lower levels of CD4 counts, even after adjusting to the absence of detectable viral load (which would indicate the use of effective antiretroviral treatment).

The authors concluded that among a population of HIV-positive military veterans with a high prevalence of risk factors for CVD, immunodeficiency (CD4 cell count below 500 cells/mm^3^) or a detectable viral load raised the risk of developing LEAD even more. Our study failed to prove this association, probably due to the limited study population (300 subjects).

Compared to previous cross sectional studies on LEAD prevalence in PLWH, our study included the largest outpatient sample. As HIV centres in Bujumbura capital deal with the regional population, our results are representative for the rural and urban HIV subjects. The limitations of our study are due to the transversal design. Other limitation of this study is the lack of a control group in order to evaluate the risk-excess of LEAD in HIV patients as compared to healthy counterparts. As inclusions were conducted in HIV-care centres, the access to non-diseased population was not feasible. Nevertheless, the increasing LEAD-risk excess with increasing HIV disease severity is an important finding of our study.

## Conclusion

In summary, the prevalence rate of LEAD in our study was very high in mid aged HIV-infected patients and strongly correlated with the latest HIV clinical stage, independently from all other CV risk factors or morbidities. Our findings underline that PLWH are at high CV risk and also point out the need for LEAD screening in HIV-infected patients.

## Data Availability

The datasets generated during and/or analysed during the current study are available from the corresponding author on reasonable request.
